# Trends in semen parameters of infertile men in South Africa and Nigeria

**DOI:** 10.1038/s41598-023-33648-4

**Published:** 2023-04-26

**Authors:** Edidiong Nnamso Akang, Chinyerum Sylvia Opuwari, Swesme Enyioma-Alozie, Lionel Wildy Moungala, Tamarapreye Emmanuel Amatu, Ibrahim Wada, Rose Ogeyi Ogbeche, Oluwatoyin Omolayo Ajayi, Mojisola Modupe Aderonmu, Olaitan Boluwatife Shote, Lateef Adekunle Akinola, Oladapo Adenrele Ashiru, Ralf Henkel

**Affiliations:** 1 Department of Anatomy, College of Medicine, University of Lagos, Idi-Araba, Lagos, Nigeria; 2 Section of Infectious Diseases, Department of Internal Medicine, Yale School of Medicine, Yale University, New Haven, Connecticut USA; 3 Department of Medical Biosciences, Faculty of Natural Sciences, University of the Western Cape, Bellville, South Africa; 4 Department of Anatomy, Baze University, Abuja, Nigeria; 5 Androcryos Andrology Laboratory, Johannesburg, South Africa; 6 Nisa Premier Hospital, Abuja, Nigeria; 7 The Bridge Clinic, Lagos, Nigeria; 8 Medical Art Center, Ikeja, Lagos Nigeria; 9 Department of Obstetrics and Gynaecology, Medison Specialist Women’s Hospital, Lagos, Nigeria; 10 Department of Metabolism, Digestion and Reproduction, Imperial College London, London, UK; 11 LogixX Pharma, Theale, Berkshire, UK

**Keywords:** Developmental biology, Physiology, Anatomy, Medical research, Urology

## Abstract

There are conflicting reports on trends of semen parameters from different parts of the globe. However, in recent times there is dearth of information on the trend in Sub-Saharan countries. Therefore, in this study we aimed at determining the trends in semen parameters in Nigeria and South Africa between 2010 and 2019. A retrospective study of semen analyses of 17,292 men attending fertility hospitals in Nigeria and South Africa in 2010, 2015 and 2019. Patients who had undergone vasectomy and those who had a pH less than 5 or greater than 10 were excluded from this study. The following variables were assessed: ejaculate volume, sperm concentration, progressive motility, total progressively motile sperm count (TPMSC), total sperm count, and normal sperm morphology. Between 2010 and 2019, significant trends of decreasing values were observed in normal sperm morphology (− 50%), and the ejaculatory volume (− 7.4%), indicating a progressive deterioration of the values in both countries. In Nigeria, there were significant decreases in progressive motility (− 87%), TPMSC (− 78%), and sperm morphology (− 55%) between 2010 and 2019 (*P* < 0.001). Spearman`s rank correlation revealed significant negative associations between age and morphology (ρ =  − 0.24, *P* < 0.001), progressive motility (ρ =  − 0.31. *P* < 0.001), and TPMSC (ρ =  − 0.32, *P* < 0.001). Patients in South Africa were younger than those from Nigeria, with also a significantly higher sperm morphology, sperm concentration, progressive motility, total sperm count and TPMSC. Our findings provide a quantitative evidence of an alarming decreasing trend in semen parameters in Nigeria and South Africa from 2010 to 2019. It also proves that astheno- and teratozoospermia are the leading causes of male infertility in these regions. In addition to this, it also shows empirically that semen parameters decrease with advancement in age. These findings are the first report of temporal trends in semen parameters in Sub-Saharan countries, necessitating a thorough investigation on the underlying factors promoting this worrisome decline.

## Introduction

Semen parameters including sperm concentration, total sperm count, sperm motility, total progressive motile sperm count (TPMSC), sperm morphology, and semen volume are essential diagnostic tools for assessing the reproductive health and fertility status of men^1^. Several studies have revealed conflicting results on the trend of semen parameters postulating there are no temporal trends and if there are then they are region dependent however, a decline has been observed since the late 1930s in western countries including the United States, Australia, and Europe^2–5^.

Aberrations in sperm parameters remain the leading cause of male factor infertility^6^. Between 1965–2015, Sengupta et al.^7^ reported an over 32% reduction in sperm concentration among European men. From the most populous countries in Asia, Adiga et al.^8^, reported 22.92%, 30.3% and 51.25% decrease in sperm motility, sperm count, and morphology respectively among Indian men. Similarly, significant decline in sperm concentration and total sperm count has been reported among 327 373 Chinese men within four decades^9^. From South America, a 23-year (1995–2018) study in Brazil also reported decline in sperm parameters^10^. Another study reported an overall 73% decline in sperm concentration in African men over a period of 50 years (1965–2015)^11^.

Globally, the declining trend in semen parameters seem to be more consistent however, in recent times there is paucity of information on the trends of semen parameters in Sub-Saharan African countries. This makes it difficult to ascertain the enormity of the burden of male factor infertility in African families. Therefore, in this study we aim at identifying the prevalence, and trends of semen parameters in Nigeria and South Africa between 2010 and 2019.

## Methods

### Study design and participants

This is a retrospective study that included the semen analysis reports of 17,292 men attending fertility hospitals in Nigeria and South Africa in 2010, 2015 and 2019. All patients who had undergone vasectomy and those who had pH less than 5 or greater than 10 were excluded from this study. Data from Nigeria were recorded from four hospitals: Medical ART Center, Maryland, Lagos; Bridge Clinic, Ikeja, Lagos; The Medison Hospital, Lekki, Lagos; Nisa Premier, Jabi, Abuja. While those from South Africa were recorded from patients attending Androcryos Andrology Laboratory, Johannesburg; Ampath Andrology Laboratory, Pretoria; and Lancet Andrology Laboratory, Pretoria.

### Semen analysis

Semen was collected by masturbation into a sterile plastic container after 2 to 7 days of abstinence and examined within one 1 h after liquefaction in accordance with the 2010 WHO guidelines (WHO, 2010). The following variables were assessed: ejaculate volume, sperm concentration, progressive motility, total progressively motile sperm count (TPMSC), total sperm count, and normal sperm morphology.

### Statistical analysis

All data were calculated using the MedCalc^®^ statistical software (Ver. 20.118; MedCalc Software Ltd, Ostend, Belgium). Data was tested for normal distribution using the Chi-squared test. Since data was not normally distributed, non-parametric tests were performed. Mann Whitney test was used to compare cumulative data between Nigeria and South Africa, whereas the Kruskal–Wallis test was used to compare differences between the different years of data retrieval (2010, 2015, and 2019). Dunn’s post-hoc analysis was carried out on samples that showed any significant difference of *P* < 0.05. Then, samples were subjected to Jonckheere-Terpstra trend test to determine if the change was stronger as the years progressed. A *P*-value of *P* < 0.05 was considered significant. In order to investigate possible influences of the patients’ age and the duration of the abstinence on normozoospermia, asthenozoospermia and teratozoospermia, a logistic regression analysis was performed. The Hosmer–Lemeshow test was used to test for the goodness of the logistic regression model. Finally, we used a pivot analysis on Microsoft office 16 Excel to determine the percentage increase/decrease between the years.

### Ethics approval and consent to participate

This study was approved by the Health Research Ethics Committee (HREC) of the College of Medicine of the University of Lagos, Lagos, Nigeria with the Ethical Approval ID: CMUL/HREC/11/21/1094. It was also approved by the Biomedical Science Research Ethics Committee of the University of Western Cape, Bellville, South Africa with the Ethics Reference number: BM19/9/7. This entire study was performed in accordance with the principles of Declaration of Helsinki. We also obtained a written informed consent to access data from each of the centers wherein the data was obtained.

## Results

Except for the time of sexual abstinence (*P* = 0.1867), significant (*P* < 0.001) differences between all other variables investigated (age, sperm concentration, normal morphology, progressive motility, total sperm count, TPMSC, and ejaculate volume) were observed between the two countries. Whereas the patients in South Africa were younger and had fewer days of abstinence than those from Nigeria, they had higher sperm morphology, sperm concentration, progressive motility, total sperm count and total progressively motile sperm count (Table 1).

**Table 1:** Summary statistics of independent variable data from Nigeria and South Africa.

Variable	Nigeria				
	n	Median (25th, 75th percentile)	n	Median (25th, 75th percentile)	*P* value^a^
Abstinence (d)	2122	4 (3.0, 4.0)	12,350	3 (3.0, 5.5)	= 0.187
Age (years)	1702	42 (34.0, 43.0)	15,595	38 (37.0, 48.0)	< 0.001
Sperm concentration (10^6^/mL)	2450	25 (15.2, 82.9)	14,847	41 (6.0, 48.0)	< 0.001
Morphology (%)	2200	5 (3.0, 7.0)	4700	5 (3.0, 20.0)	< 0.001
Progressive motility (%)	2514	15 (25.0, 49.0)	1402	40 (3.0, 52.0)	< 0.001
Total sperm count (10^6^)	2448	48.7 (35.2, 227.5)	13,462	104.34 (12.0, 110.0)	< 0.001
TPMSC (10^6^)	2425	6.3 (9.4, 95.3)	1402	38.48 (0.4, 29.0)	< 0.001
Volume (mL)	2595	2 (1.9, 3.8)	13,553	2.6 (1.6, 3.0)	< 0.001

^a^Mann-Whitney test.

Table 2 presents the results for the two countries, Nigeria and South Africa as well as the combined data from both countries between 2010 and 2019. A significant decline in sperm concentration (*P* = 0.003), normal morphology (*P* < 0.001), total sperm count (*P* < 0.001), TPMSC (*P* < 0.001), and volume (*P* < 0.001) was noted for South Africa, with a significant increase in progressive motility (*P* < 0.001). A significant declining trend was also noted for sperm concentration (*P* = 0.01217), normal morphology (*P* < 0.001), Total sperm count (*P* < 0.001), TPMSC (*P* < 0.001) and volume (*P* < 0.001), while progressive motility increased (*P* < 0.001) in South Africa. In addition, normal morphology (*P* < 0.001), progressive motility (*P* < 0.001), TPMSC (*P* < 0.001) decreased significantly, while sperm concentration, total sperm count, and semen volume remained unchanged (P > 0.05) in Nigeria. Furthermore, a declining trend was only noted for normal morphology (*P* < 0.001), progressive motility (*P* < 0.001), and TPMSC (*P* < 0.001). The combined data of both countries showed a significant decrease in normal morphology (*P* < 0.001), progressive motility (*P* < 0.001), TPMSC (*P* < 0.001) and semen volume (*P* < 0.001). Similarly, a significant declining trend was observed for normal morphology (*P* < 0.001), progressive motility (*P* < 0.001), TPMSC (*P* < 0.001) and semen volume (*P* < 0.001) in the combined date.

**Table 2:** Comparison of the independent variables from Nigeria and South Africa and the combination of both countries.

Variables	Country	Year	n	Median (25th, 75th percentile)	*P* value
Sperm concentration (10^6^/mL)	South Africa	2010	4594	43.2 (16,23)	= 0.003
		2015	5334	40 (15, 27)	
		2019	4919	40 (15, 20)	
	Nigeria	2010	915	23 (7, 43)	= 0.133
		2015	1028	27 (7, 49)	
		2019	507	20 (5, 48)	
	Combined	2010	5509	38 (14, 79)	= 0.075
		2015	6362	38 (14, 73.6)	
		2019	5426	38 (14, 78.8)	
Normal morphology (%)	South Africa	2010	1237	9 (6,13)	< 0.001
		2015	1666	4 (3, 6)	
		2019	1797	4 (3,5)	
	Nigeria	2010	815	5 (1,11)	< 0.001
		2015	860	10 (4, 40)	
		2019	525	5 (5, 15.1)	
	Combined	2010	2052	8 (4,13)	< 0.001
		2015	2526	5 (3, 9)	
		2019	2322	4 (3, 5)	
Progressive motility (%)	South Africa	2010	1305	38 (25, 48)	< 0.001
		2015	N/A	N/A	
		2019	97	50 (40, 51.2)	
	Nigeria	2010	937	38 (10, 57)	< 0.001
		2015	1053	15 (2.8, 50)	
		2019	524	5 (0, 12.4)	
	Combined	2010	2242	38 (20,50)	< 0.001
		2015	1053	15 (2.8, 50)	
		2019	621	5 (0, 40)	
Total sperm count (%)	South Africa	2010	3268	117.62 (38.4, 253)	= 0.001
		2015	5330	100 (34.6, 216)	
		2019	4864	102 (34, 223.1)	
	Nigeria	2010	914	45 (12, 104.4)	= 0.08
		2015	1027	51 (12, 120)	
		2019	507	46 (12, 104.8)	
	Combined	2010	4182	94.4 (29.4, 217)	= 0.186
		2015	6357	90 (30,200)	
		2019	5371	93.4 (30, 210)	
TPMSC (10^6^)	South Africa	2010	1305	41.99 (12, 98.7)	< 0.001
		2015	N/A	N/A	
		2019	97	1.49 (0, 36)	
	Nigeria	2010	905	10.4 (1.9, 38.2)	< 0.001
		2015	1014	6.2 (0.2, 32)	
		2019	506	2.3 (0, 10.7)	
	Combined	2010	2210	25.5 (4.8, 73.6)	< 0.001
		2015	1014	6.2 (0.2, 32)	
		2019	603	2.2 (0, 13)	
Volume (mL)	South Africa	2010	3280	2.9 (2, 4)	< .001
		2015	5378	2.7 (1.8, 3.8)	
		2019	4895	2.5 (1.9, 3.5)	
	Nigeria	2010	1005	2 (1.5, 3)	= 0.188
		2015	1065	2 (2,3)	
		2019	525	2 (2,3)	
	Combined	2010	4285	2.7 (1.9, 3.8)	< 0.001
		2015	6443	2.5 (1.8, 3.5)	
		2019	5420	2.5 (1.9, 3.5)	

Over the years, as observed from 2010 to 2019, significant trends of decreasing values are observed for, sperm concentration (− 0.23%), normal sperm morphology (− 50%), total sperm count (− 1.1%) and ejaculatory volume (− 7.4%) indicating a progressive deterioration of the values, combining data from Nigeria and South Africa. Due to the small data from South Africa in 2019 on progressive motility and TPMSC a combined assessment of both countries was not done (Table 3).

**Table 3:** Summary of percentage increase/decrease of data between 2010 and 2019.

Variables	Nigeria (%)	South Africa (%)	Combined (%)
Sperm morphology (%)	0	− 55	− 50
Progressive motility (%)	− 86.8	ND	ND
Sperm concentration (10^6^/mL)	− 13	− 7.4	− 0.23
Total sperm count (10^6^)	2.2	− 0.1	− 1.1
TPMSC (10^6^)	− 77.9	ND	ND
Volume (mL)	0	− 13.8	− 7.4

Percentage change was calculated using the median values.

*ND* No data due to small sample size.

In addition, some changes were noted in the trends for Nigeria and South Africa between 2010 and 2019. In Nigeria, a decline was observed in sperm concentration (− 13%), progressive motility (− 86.8%), and TPMSC (− 77.9%), with an increase in total sperm count (2.2%), while there were no changes observed for morphology, and ejaculatory volume. In South Africa, a decline in sperm concentration (− 7.4%), normal sperm morphology (− 55%), total sperm count (− 0.1%), and ejaculatory volume (− 13.8%) was observed. Data for progressive motility and TPMSC is not available for South Africa due to small sample size (Tables 3).

Spearman`s rank correlation for abstinence and age with semen parameters from Nigeria and South Africa showed weak associations between the duration of abstinence and all semen parameters, whereas there were moderate negative correlations between age and morphology (ρ =  − 0.24, *P* < 0.001), progressive motility (ρ =  − 0.31, *P* < 0.001), and TPMSC (ρ =  − 0.32, *P* < 0.001). The other parameters showed weak negative correlations (Table 4).

**Table 4:** Spearman rank correlations for various parameters in Nigeria, South Africa and for the data of these countries combined.

Country			Abstinence (d)	Age (years)	Sperm Conc. (10^6^/mL)	Normal morphology (%)	Prog. motility (%)	Total sperm count (10^6^)	TPMSC (10^6^)	Volume (mL)
Nigeria	Abstinence (d)	r		0.1	0.038	− 0.121	0.008	0.084	0.026	0.147
		*P*		< 0.001	0.0828	< 0.001	0.7294	< 0.001	0.2361	< 0.001
	Age (years)	r	0.1		0.018	− 0.084	− 0.137	− 0.006	− 0.094	− 0.084
		*P*	< 0.001		0.4869	< 0.001	< 0.001	0.8187	< 0.001	< 0.001
South Africa	Abstinence (d)	r		− 0.058	0	− 0.011	0.002	0.022	0.112	0.067
		*P*		< 0.001	0.9575	0.7055	0.9546	0.019	< 0.001	< 0.001
	Age (years)	r	− 0.058		0.004	− 0.068	− 0.064	− 0.04	− 0.087	− 0.091
		*P*	< 0.001		0.6082	0.0168	0.0187	< 0.001	0.0014	< 0.001
Nigeria and South Africa combined	Abstinence (d)	r		− 0.036	0.004	− 0.067	0.08	0.03	0.133	0.079
		*P*		< 0.001	0.6542	< 0.001	< 0.001	< 0.001	< 0.001	< 0.001
	Age (years)	r	− 0.036		− 0.025	− 0.243	− 0.309	− 0.074	− 0.316	− 0.109
		*P*	< 0.001		0.0012	< 0.001	< 0.001	< 0.001	< 0.001	< 0.001

Spearman`s rank correlation for abstinence and age with semen parameters from Nigeria and South Africa showed weak associations between the duration of abstinence and all semen parameters, whereas there were moderate negative correlations between age and morphology (ρ =  − 0.24, *P* < 0.001), progressive motility (ρ =  − 0.31, *P* < 0.001), and TPMSC (ρ =  − 0.32, *P* < 0.001). We also observed weak correlations between abstinence and age with semen parameters in Nigeria, and in South Africa. The data are depicted in Table 4.

To analyze the influence of the independent variables age, abstinence and ejaculate volume on the clinically important classifications normozoospermia, asthenozoospermia and teratozoospermia, a logistic regression was calculated. For normozoospermia, the overall fit of the model was significant (*P* = 0.0019) with good goodness of the fit (Hosmer–Lemeshow test: *P* = 0.3320) and a significant (*P* = 0.0015) effect of age while abstinence and ejaculate volume had no effect (Table 5). When analysing separately for the two countries, the model was also significant. However, the effect for age was only significant for South Africa, while a significant influence was observed for the ejaculate volume.

**Table 5:** Logistic regression analyze the influence of the independent variables age, abstinence and ejaculate volume on the clinically important classifications normozoospermia, asthenozoospermia and teratozoospermia.

Country	Variable	Coefficient	Std. Error	*P*	
Normozoospermia					
South Africa/Nigeria combined	Overall fit			**0.0019**	
	Age (yrs)	− 0.0099	0.0031	**0.0015**	
	Abstinence (d)	− 0.0093	0.0058	0.1096	
	Volume (mL)	0.0162	0.0137	0.2377	
South Africa	Overall fit			**0.0074**	
	Age (yrs)	− 0.0116	0.0035	**0.0011**	
	Abstinence (d)	− 0.0062	0.0077	0.4168	
	Volume (mL)	0.0064	0.0144	0.6562	
Nigeria	Overall fit			**0.0492**	
	Age (yrs)	− 0.0012	0.0076	0.8733	
	Abstinence (d)	− 0.0149	0.0112	0.1820	
	Volume (mL)	0.1254	0.0560	**0.0252**	
Asthenozoospermia					
South Africa/Nigeria combined	Overall fit			< **0.001**	
	Age (yrs)	0.0933	0.0068	< **0.001**	
	Abstinence (d)	− 0.0271	0.0108	**0.0127**	
	Volume (mL)	− 0.1024	0.0287	< **0.001**	
South Africa	Overall fit			0.0879	
	Age (yrs)	0.0159	0.0081	**0.0497**	
	Abstinence (d)	− 0.0248	0.0145	0.0872	
	Volume (mL)	0.0007	0.0348	0.9843	
Nigeria	Overall fit			**0.0024**	
	Age (yrs)	0.0592	0.0814	0.4671	
	Abstinence (d)	19.1660	9137.9881	0.9983	
	Volume (mL)	1.3489	1.1296	0.2324	
Teratozoospermia					
South Africa/Nigeria combined	Overall fit				< **0.001**
	Age (years)	0.0352	0.0054		< **0.001**
	Abstinence (d)	0.0087	0.0077		0.2595
	Volume (mL)	− 0.1529	0.0365		< **0.001**
South Africa	Overall fit				0.2112
	Age (years)	0.0215	0.0119		0.0711
	Abstinence (d)	− 0.0034	0.0207		0.8705
	Volume (mL)	− 0.0513	0.0533		0.3364
Nigeria	Overall fit				< **0.01**
	Age (years)	0.0125	0.0070		0.0750
	Abstinence (d)	0.0216	0.0149		0.1473
	Volume (mL)	− 0.1628	0.0528		**0.0021**

Significance values are in bold.

For asthenozoospermia, a good significant fit (overall fit: *P* < 0.001; Hosmer–Lemeshow test: 0.7231) was detected. Separate analysis for South Africa and Nigeria resulted in significant overall fits of the model (Table 5), but only a significantly fitting model for Nigeria and not for South Africa. While the impact of age was just significant for the South African cohort, no significant impact of the independent variables was observed (Table 5).

For teratozoospermia, the model for the combined data from South Africa and Nigeria was significant with good goodness of fit (*P* < 0.001; Hosmer–Lemeshow test: 0.5327). However, in the separate analysis, the model was only significant for Nigeria with the ejaculate volume having a significant impact (Table 5).

## Discussion

It is now known that 50% of infertility cases are male factor related and this is largely determined by semen parameters in reference to WHO reference material^1^. Previous studies have demonstrated that semen parameters may change over time and vary based on different geographic regions^12–14^. It is not completely clear if there are temporal trends in semen parameters especially in Sub-Saharan African countries. Hence, this study aimed to identify the trends in semen parameters in infertile men in South Africa and Nigeria.

By analysing the independent variables utilized in this study we observed a significant declining trend in normal morphology, progressive motility, and total progressive motile sperm count (TPMSC) between 2010 and 2019 among the infertile men in South Africa and Nigeria. With a high decline of 86.8% of progressive motile sperm, and 77% of TPMSC in Nigeria and 55% of normal morphology in South Africa, this report stands significantly higher than the ~ 11% decline in total motile sperm count observed in the United states of America^15^, the 22.5% decline in motility reported in India^8,16^, and the 38.2% decline in China^17^. Though it is difficult to know the state of progressive motility in South Africa, the alarming decline in progressive motility observed in Nigeria seems to be the major reason for the increased male infertility observed among the men attending fertility clinics. Sperm motility plays a central role in both natural and several forms of assisted fertilization since only progressive sperm cells will swim through the vagina, cervical mucous, tortuous endometrium, and ultimately arrive at the ampullary site of the fallopian tube for fertilization^18–20^. This study showed that progressive sperm motility decreased from 38 to 5% between 2010 and 2019. This is far below the lower limit (40%) recommended by the World Health Organization for progressive motility^1^, meaning more patients are suffering from asthenozoospermia from these parts of the globe. Asthenozoospermia may be due to a couple of factors including genetic, molecular, and environmental factors^20–22^.

In our study, between 2010 and 2019 we observed a decline from 8 to 4% in normal sperm morphology which is below the lower limit of semen analysis for healthy men^1^. Our findings are in corroboration with the southern Tunisian report of a decline in normal morphology over a 12 year period^23^. Though abnormal sperm morphology does not seem to affect fertilisation and embryo development from intracytoplasmic sperm injection (ICSI) procedures, it is associated with higher implantation and pregnancy failures^24,25^. Although the role of sperm morphology in natural and assisted pregnancy is still controversial our findings infer that perhaps there is increased alterations on spermiogenesis which may have genetic or epigenetic undertones^26,27^.

A comparison of the data collected from South Africa and Nigeria revealed that the number of days of sexual abstinence by patients, and normal sperm morphology were similar in both countries, while the patients’ age was higher in Nigeria compared to South Africa. The reason for this is not clear but we may allude to the low health insurance coverage in Nigeria which has only 3% of its populace on health insurance as compared to about 20% in the South Africa populace, making it financially burdensome for young couples to seek assisted reproductive care^28–30^.

In addition, we recorded a reduction in the following semen parameters (sperm concentration, progressive motility, total sperm count, total progressive motile sperm count (TPMSC) and semen volume) with increase in age among Nigerian patients compared to South African samples which came from younger men. This is in concordance with reports that aging plays a major role in decreasing semen quality^31,32^. This is buttressed by our correlation analysis, which observed a significant inverse proportional relationship between age and all the semen parameters analysed.

A number of factors could be associated with aging and poor semen quality, but they could be broadly divided into two categories: First, degenerative changes in the reproductive tract due to progressive decline in cellular function. An increased disorientation in centrosomes which should ensure asymmetric division of germline stem cells has been observed in aged testis thereby distorting the proportion of self-renewing spermatogonia to differentiating spermatogonia thus, causing a decline in spermatogenesis^33^. Oxidative stress has also been linked to advanced paternal aging causing increased inflammation and endocrine dysfunction ultimately impeding spermatogenesis^34^. In addition, there is marked reduction of Leydig cells with age, increased testicular atrophy and Sertoli cells^35,36^. Secondly, the older one gets, the more prone they are to exogenous toxicants including diseases, lifestyle, environment, and climate change that could be detrimental to the reproductive tract. For instance, older ones are more likely to have smoked, or consumed alcohol for longer which adversely affects semen quality^37–39^. Some therapies for chronic diseases such as human immunodeficiency virus (HIV) and the disease itself reduces the seminal fluid, sperm motility and increases sperm DNA fragmentation^21^, High temperatures also exacerbate the production of reactive oxygen species in testis inducing testicular germ cell apoptosis^40,41^. In addition, benign prostatic hyperplasia is also associated with ageing which may result in decrease in ejaculated semen volume^42,43^.

This study revealed that in South Africa, a decline was noted for sperm concentration, normal sperm morphology, total sperm count, and volume with an increasing trend for progressive motility. Perhaps this was due to the very small data available in 2019 compared to that of 2010. Nigeria, which is 100% black dominated country, had declining trend in progressive motility and TPMSC. Nigeria also had significantly lower sperm concentration compared to South Africa which is of mixed races. It is not clear if our findings are of any racial correlations, however, Auger et al., reported that black men had significantly lower sperm concentrations than white men and Hispanics^44^. Furthermore, exposure to the pesticide, DDT [1,1,1-trichloro-2,2-bis (chlorodiphenyl)ethane] and its main metabolite, p,p′-dichlorodiphenyl-dichloroethylene (p,p′-DDE) has been associated with the declining trend of semen quality in malaria stricken parts of African countries^45^. In addition, environmental pollutants, such as crude oil spillage, and gas flaring increases the oxidative stress levels thereby causing a decline in semen quality^46,47^.

## Conclusions

This study reports observations of a rapid decreasing trend in semen parameters among men visiting fertility clinics in South Africa and Nigeria between 2010 and 2019. This alarming increase in astheno- and teratozoospermia is the leading cause of male infertility in Nigeria and South Africa respectively. This study also identifies ageing as a factor associated with the decreased semen parameters. Our findings may give some insight into the temporal trends in semen parameters specifically in Sub–Sahara Africa and suggest further studies to investigate the other causes of the increasing male factor infertility. Furthermore, trends of semen parameters in different regions of the world need to be investigated, causes identified and solutions proffered for this increasing problem challenging men`s reproductive health.

### Limitation

The fact that only semen analysis from two African countries, Nigeria and South Africa, were analyzed can be seen as a limitation of this study. In addition, considering that only patients attending an ART or andrology center were analyzed, no information about the general population could be provided. Furthermore, hormone profiles and demographic and clinical aspects of the patients were not included within the scope of this study and are therefore recommended for future studies.

## Data Availability

All data generated or analysed during this study are included in this published article.
